# Hyperoxia retards growth and induces apoptosis and loss of glands and blood vessels in DMBA-induced rat mammary tumors

**DOI:** 10.1186/1471-2407-7-23

**Published:** 2007-01-30

**Authors:** Anette Raa, Christine Stansberg, Vidar M Steen, Rolf Bjerkvig, Rolf K Reed, Linda EB Stuhr

**Affiliations:** 1 Department of Biomedicine, University of Bergen, Norway; 2 Department of Clinical Medicine, University of Bergen, Norway; 3 Center for Medical Genetics and Molecular Medicine, Haukeland University Hospital, Bergen, Norway; 4 Norlux Nevro-Oncology, Centre Research Public Santé, Luxenbourg

## Abstract

**Background:**

This study investigated the effects of hyperoxic treatment on growth, angiogenesis, apoptosis, general morphology and gene expression in DMBA-induced rat mammary tumors.

**Methods:**

One group of animals was exposed to normobaric hyperoxia (1 bar, pO_2 _= 1.0 bar) and another group was exposed to hyperbaric hyperoxia (1.5 bar, pO_2 _= 1.5 bar). A third group was treated with the commonly used chemotherapeutic drug 5- Fluorouracil (5-FU), whereas animals housed under normal atmosphere (1 bar, pO_2 _= 0.2 bar) served as controls. All treatments were performed on day 1, 4, 7 and 10 for 90 min. Tumor growth was calculated from caliper measurements. Biological effects of the treatment, was determined by assessment of vascular morphology (immunostaining for von Willebrandt factor) and apoptosis (TUNEL staining). Detailed gene expression profiles were obtained and verified by quantitative rtPCR.

**Results:**

Tumor growth was significantly reduced (~57–66 %) after hyperoxic treatment compared to control and even more than 5-FU (~36 %). Light microscopic observations of the tumor tissue showed large empty spaces within the tissue after hyperoxic treatment, probably due to loss of glands as indicated by a strong down-regulation of glandular secretory proteins. A significant reduction in mean vascular density (30–50%) was found after hyperoxic treatment. Furthermore, increased apoptosis (18–21%) was found after hyperoxic treatment.

**Conclusion:**

Thus, by increasing the pO_2 _in mammary tumor tissue using normobaric and moderate hyperbaric oxygen therapy, a significant retardation in tumor growth is achieved, by loss of glands, reduction in vascular density and enhanced cell death. Hyperbaric oxygen should therefore be further evaluated as a tumor treatment.

## Background

Growth of solid tumors depends on adequate supply of oxygen and nutrients. There are, however, marked differences in the vascular network in the different regions of the tumor. In the centre, there is typically a hypoxic milieu due to structural and functional vessel disturbances (perfusion- and diffusion-limited O_2 _delivery), while in the periphery there is generally a denser vascular network with subsequent improved blood flow. While normal tissue can compensate for such an O_2 _deficiency by raising the blood flow, large tumor areas cannot adequately counteract the restriction in O_2 _supply and therefore develop hypoxia. Thus, the HbO_2 _saturation is significantly lower in tumors than in normal surrounding tissue, with a gradual reciprocal decrease as the tumor increases in size [1,2].

It is now widely accepted that hypoxia promotes tumor growth, angiogenesis and reduce the effect of chemo- and radiation- therapy [3-6]. We might therefore expect that an increase in the oxygen-content in tumor tissue might have the opposite effect.

Hyperbaric oxygen treatment (HBO) offers one possibility to increase the oxygen content in various tissues [7-10]. The use of HBO in cancer therapy has been aimed at improving the radiation response in solid tumors [10] as well as to improve healing of normal tissue after radiation injury [11]. The increase in tissue pO_2 _during and after HBO treatment is due to enhanced transport of soluble oxygen. The physically solved oxygen at normobaric air pressure is approximately 0.3 ml O_2_/l00 ml blood, with a corresponding HbO_2 _of approximately 21 ml/100 ml blood. By breathing 100% oxygen at normobaric pressure, the amount of physically soluble oxygen increases 6 times (1.8 ml O_2_/100 ml blood). If the atmospheric pressure is elevated to 3 bar in the presence of 100% O_2_, the amount of oxygen delivered to the tissue would increase to 6.0 ml O_2_/100 ml blood, which is even sufficient to support resting tissue independent of the O_2 _contribution from hemoglobin [12]. When oxygen is in solution, it can reach physiologically obstructed areas that are inaccessible to the HbO_2_-containing red blood cells. In line with this, several investigators have measured a significant delay in washout (15–60 min) of the pO_2 _in different tumors after HBO treatment [3,7-9].

Due to the apparent link between blood supply, oxygenation and tissue growth, it has for a long time been a misconception that HBO *per se *could have a tumor- promoting effect. There are now several lines of evidence showing that this is not the case [13]. In a rat model of dimethyl-α-benz-anthracene (DMBA)-induced mammary adenocarcinomas, we have recently demonstrated a significant decrease in mammary tumor size after repeated HBO treatment at 2 bar for 90 minutes [14]. These paradoxical data indicate that an increase in the delivery of physically dissolved O_2 _in the tumor tissue by hyperbaric hyperoxic treatment may suppress its growth.

The present study was initiated to see if 1.5 bar (pO2 = 1.5) as well as pure oxygen at normal atmospheric pressure (1 bar, pO_2 _= 1.0) would have a similar effect. The aim of the present study was therefore to find the least pressure gradient that gave a therapeutic effect on tumor growth. Therapeutic efficacy was determined by assessing tumor growth, angiogenesis, apoptosis, general morphology and gene expression profiling.

## Methods

### Animal model of mammary tumors

Female Sprague-Dawley (Møllegård, Denmark) rats (n = 35) were used. Mammary tumors (adenocarcinomas) were induced by dimethyl-α-benz-anthracene (DMBA) dissolved in olive oil and given to the 7 week rat by gavage at a dose of 16 mg. The experiments were performed when the rats were 13–15 weeks old, having reached a body weight of 250–300 g and developed tumors along the mammary crest. Tumor size was measured externally by calipers on day 1, 4, 7, and 11 and estimated according to the following formula: π/6 · (a)^2^(b), where a is the shortest transversal diameter and b is the longest transversal diameter. The examinations were performed by the same person, with no knowledge about the various exposures of the rats. All measurements were performed during isofluran (Shering-Plough AS, Narum, Denmark) and N2O anesthesia (Ohmeda: BOC Health Care, Weast-Yorkshire, England). The study was approved by the Norwegian Committee for Animal Research (Oslo, Norway).

### Experimental groups and treatment design

Four separate groups of rats were studied (for details see Table 1).

### Hyperbaric chamber and oxygen exposure procedure

130 l hyperbaric chamber with an internal diameter of 50 cm was used. For inspection and video supervision there are two windows in the chamber wall. Penetrators for gas inlet and outlet run through the chamber's end wall. The chamber and rat cage were cleaned and degreased (96–100% ethanol) before starting exposure with pure oxygen. All electrical installations were disconnected. The rats were placed in litter-free cages (590 × 385 × 200 mm) during HBO exposure. All rats were showered slightly with water prior to entering the pressure chamber, due to the danger of fire in a pure oxygen atmosphere. Four petridishes with water were placed on the chamber floor to additionally humidify the atmosphere. To initiate the treatment, 100% oxygen was introduced into the chamber as air was simultaneously flushed out. The oxygen concentration in the chamber was monitored continuously by an oxygen cell (C3, Middelsborough, England). When oxygen was above 98%, the rats of group 3 were kept at this level for 90 min, while in group 4 the chamber was pressurized with oxygen over approximately 2 min to 1.5 bar, and this pressure was maintained throughout 90 min. During the 90 min period, the chamber was flushed twice for 5 min (at 30 and 60 min) with pure oxygen in both series. The temperature was held at approximately 22°C and the humidity at approximately 100%. The CO_2 _content was kept low by use of a scrubber material (Sodasorb, Molecular Products United Drug Co Ltd, Essex, UK) placed inside the chamber floor.

### Morphological analyses

The animals were sacrificed with pentobarbital during isofluran and N2O anesthesia, and the tumors were dissected out (one tumor from each rat). One part of the tumor was fixed in 4% buffered formalin, processed and embedded in paraffin. Tissue sections of each specimen were stained using Harris Haematoxylin and Eosin (H & E, Merck, Damstadt, Germany). The sections were analyzed with regards to apoptosis (TUNEL assay) and blood vessel density (von Willebrand factor). Indirect immunohistochemistry was performed by the EnVisison™ + System, horse-radish peroxidase and 3'3'-diaminobenzidine (DAB) (DAKO, Glostrup, Denmark) method, as described in the manufacturer's protocol.

### TUNEL staining

Apoptosis was examined by the terminal transferase-mediated dUTP nick end-labeling (TUNEL) method (Boehringer Mannheim, Mannheim, Germany), performed according to the manufacturers recommendations. The DNA strand breaks in apoptotic cells are labelled by attaching biotin-or digoxygenin conjugated dUTP in a reaction catalyzed by exogenous terminal deoxynucleptidyl transferase (TdT-assay) or DNA polymerase. For antigen retrieval the slides were put into citrate buffer (0.1 M, pH6.0) and then microwaved for 5 min. The slides were washed in PBS before terminal deoxynucleotidyl transferase was applied to each slide and incubated for 1 hr at 37°C in a humidified chamber. A stop/washing buffer was used before applying the converter POD (anti-fluorescein antibody conjugated with peroxidase as reporter enzyme) for 30 min. Diaminobenzidine (DAB) was used as a chromogen and the slides were counterstained with hematoxylin. The density of apoptotic cells per mm^2 ^of the tumor viable zone was determined using a counter grid (10 random vision fields × 40). The percentage of apoptotic cells is expressed as a percentage of total cells.

### Von Willebrand factor

For detection of vascular endothelial cells we stained for Von Willebrand factor. The rabbit anti-human polyclonal antibody against Von Willebrand factor (DAKO) was diluted in TrisBSA to a 1:250 and than to a 1:500 dilution. The tissue sections were incubated for 45 min and 75 min respectively with the primary antibody, washed three times for 5 min in TBS and further incubated with anti-rabbit IgG (DAKO Cytomation Envision Kit, Labelled Polymer – HRP Anti Rabbit) for 35 min. All incubations were performed in a humidity chamber. The tissue was then washed three times with PBS. Detection was carried out using a DAB chromogen, which resulted in a positive brown staining. Harris Haematoxylin (Merck, Damstadt, Germany) was used for nuclear counterstaining. Negative control slides were obtained by omitting the primary antibody. Quantification of the microvessels was performed in 10 consecutive fields in both the center and in the periphery and averaged as vessels/mm^2^. Approximately 50 blood vessels in both the periphery and central part of the tumor were randomly selected for diameter measurements.

All the specimens were examined in a Nikon light microscopy (THP Eclipse E600, Nikon Corporation, Tokyo, Japan) and the images captured with a Nikon Digital Camera (DXM 1200F, Nikon Corporation, Tokyo, Japan). The image processing and analysis system LUCIA, version 4.8 (Laboratory Imaging Ltd, Prague, The Check Republic) was used.

### RNA extraction and microarray-based expression analysis

Specimens of tumor tissue (approximately 2 × 1 × 0.3 cm) were immersed in ice cold RNA-later solution (Ambion, Foster City, CA), and kept at 4°C over night. On the following day, the tissue tubes were frozen at -20°C until RNA isolation. The tumor tissue samples were homogenised by a Kinematica Polytron homogenizer. Total RNA was extracted using the QIAGEN RNAeasy Lipid kit, and the amount and quality of the extracted RNA was measured by the NanoDrop^® ^ND-1000 Spectrophotometer and the Agilent 2100 Bioanalyzer. Purified total RNA was stored at -80°C until use.

Twenty microgram total RNA was reversely transcribed and fluorescently labelled using the Agilent Fluorescent Direct Label Kit (G2557A), according to the manufacturer's instructions. To screen for gene expression changes in mammary tumors as a result of hyperoxic exposure, competitive hybridisations of samples from the treated tumors (Group 3, 100% O_2_, 1 bar) against the untreated tumors (normal air, 1 bar) were performed, where four hyperoxia-treated tumor samples were each compared to a pool of ten control tumors. Test samples were labelled with Cy5 and control samples labelled with Cy3. After the labelling reactions, the dye-incorporation ratio was determined using a spectrophotometer, showing ratios within 10 to 20 pmol per μg cDNA. The amount of the Cy-labelled cDNA was checked by measuring its optical density in the NanoDrop^® ^ND-1000 Spectrophotometer, at OD 260/280 nm for DNA purity and at OD 550 nm and 650 nm for dye incorporation. The amount of cDNA output was 1–2 μg and the dye incorporation was about 5% for Cy3 and 3% for Cy5.

All hybridisations were carried out on Agilent 22 k Rat Oligo Microarrays (G4130A), with 22,575 60-mer probes representing over 20,000 rat genes, ESTs and EST clusters. For hybridisation, the Agilent 60-mer oligo microarray processing protocol (V 4.1, SureHyb 22 k chamber/SSC wash) was strictly followed. Briefly, the entire labelling reactions for Cy3-labelled control samples and Cy5-labelled test samples were combined and applied to the microarray together with control targets and hybridisation buffers provided by the Agilent *in situ *hybridization plus kit (5184–3568). The microarrays were then incubated in an Agilent hybridisation oven (G2545A) with rotation for 17 hours at 60°C. On the following day, microarrays were washed and immediately dried using an ultra pure filtered N_2_stream. All operations were in accordance with the manufacturer's instructions.

After drying, the slides were scanned for fluorescence signals by the Agilent G2565AA Microarray Scanner with a pixel size of 10 nm. The TIFF images generated by the scanning process were imported into GenePixPro 5.0 software (Axon Instruments Inc) for primary data analysis. Control spots and spot artifacts were flagged in GenePix for subsequent filtering and background-corrected signal intensity values were imported into the J-Express Pro V2.6 software (MolMine, Bergen, Norway)[15]. Flagged spots were filtered and the red versus green signal intensities of each spot were adjusted by global normalization to reduce the impact of dye bias in the data sets.

Identification of genes with significant differential expression in hyperoxic exposed tumors was carried out using 'significance analysis of microarrays' (SAM) (16). The normalized dataset from J-Express was imported into the TM4 Microarray Software Suite Multi Experiment Viewer 3.1 (MeV) (TIGR, US). Signal intensities in all HBO treated 'test' samples were compared to those of the untreated 'control' samples. The analysis threshold was set to a highly conservative false discovery rate of zero.

### Real-Time PCR Analysis

Total RNA was extracted, quality controlled and stored as described above. From each of the samples, 50 ng RNA was reverse transcribed to cDNA using TaqMan RT reagents (Applied Biosystems, Foster City, CA). The final concentrations of the reagents were as follows: 1 × TaqMan RT buffer, 5.5 mM MgCl_2_, 2 mM dNTP mixture, 2.5 mM random hexamers, 0.4 U/μl RNase inhibitor and 1.25 U/μl Multiscribe reverse transcriptase in RNase-free water to a total volume of 50 μl. The reaction mix was incubated at 25°C for 10 min (primer annealing), 48°C for 30 min (synthesis) and 95°C for 5 min (enzyme inactivation). The resulting cDNA samples were stored at -20°C. All subsequent real-time PCR experiments were performed on an ABI Prism 7900 HT sequence detector system using 384-well plates. The PCR reaction solution for *parotid secretory protein (Psp), prolactin induced protein (Pip) and common salivary protein 1 (Csp1) *contained 1 μl 20 × TaqMan Gene Expression Assays (Applied Biosystems) and 10 μl 2 × TaqMan Universal PCR Master Mix. The acidic ribosomal protein P0 (P0) reaction solution contained 10 μl 2 × SYBR green (Medprobe), together with forward and reverse primers (Sigma-Aldrich) in a final concentration of 300 nM. All PCR reactions contained 4 μl of cDNA reaction mix and RNasefree water to a total volume of 20 μl. The real-time PCR was run as follows: 50°C for 2 min (UNG incubation) and 95°C for 10 min (AmpliTaq Gold activation), followed by 40 cycles of 95°C for 10 s and 60°C for 1 min. For each sample, the gene expression was quantified by the standard curve method and normalized against the expression of the ribosomal protein P0 gene. The standard curve consisted of five points that were obtained by a two-fold serial dilution of control RNA, starting out at 250 ng, together with a 'non-template' control; all performed in triplicate. P0 was preferred as the endogenous control over GAPDH and b-actin, which both gave qualitatively similar results in pilot experiments (data not shown).

### Statistics

Results were expressed as means ± SD unless indicated otherwise. Differences between groups were assessed by unpaired, two-tailed Student's t-test or the Mann-Whitney U test. P < 0.05 was considered significant.

## Results

### Tumor growth

**Figure 1:**
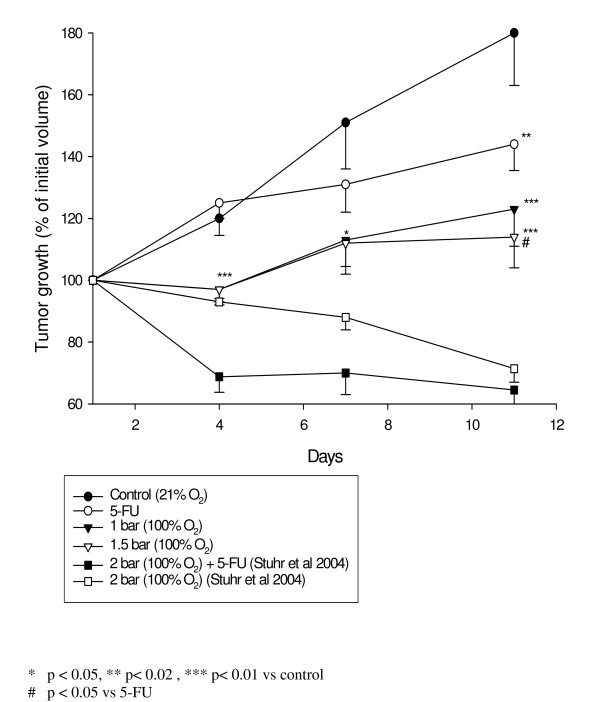
The effect of normoxic and hyperoxic treatment on tumor growth compared to controls and 5-FU treated. Treatments were given on day 1, 4, 7 and 10. Values represent means ± SE.

A total of 36 tumors were studied (one from each rat). The tumor size ranged from 1.2 to 2.3 cm^3 ^prior to the start of the experiments (Day1). The marked increase in tumor size found in controls (group 1, n = 10) over a period of 11 days (p < 0.001), was reduced by administering 5-FU to the animals (group 2, n = 10, p < 0.02). A further reduction in tumor size was observed at day 11 after both 1.0 bar (group 3, n = 8) and 1.5 bar (group 4, n = 8) hyperoxia treatment as compared to controls (p < 0.01) and 5-FU treated rats (p < 0.05). Our previous study at 2 bar, 100% O_2 _significantly attenuated tumor growth in the same tumor model (included in Fig. 1) [14]. Taken together these results indicate a reduction in growth that is dose- dependent on pO_2_.

### Down-regulation of glandular secretory proteins

Since these results confirmed our previous findings that hyperoxia might attenuate the growth of rat mammary tumors, we decided to perform a global gene expression profiling to search for differentially expressed genes and their biological function. By comparison of global gene expression level in 5 hyperoxia treated tumors with a pool of 10 control tumors, we notably observed a small cluster (n = 5) of genes involved in glandular secretion that were all strongly down-regulated by the exposure of the tumors to 100% O_2 _(Table 2). These proteins included *parotid secretory protein (Psp), common salivary protein 1 (Csp1) and prolactin induced gene (Pip), cystein-rich secretory protein (crisp-1) and proline rich proteoglycan 2 (Prpg2)*. All these genes displayed moderate to high expression levels in the control tumors, but after a total of 6 hours of hyperoxic treatment their expression was dramatically diminished, with a down-regulation ranging from 5–33 times in the individual tumors. To verify the microarray results, we used quantitative real- time PCR, which confirmed that the expression of Psp, Pip and Csp1 was markedly diminished in the hyperoxia-exposed tumors (Table 2).

### General morphology

**Figure 2:**
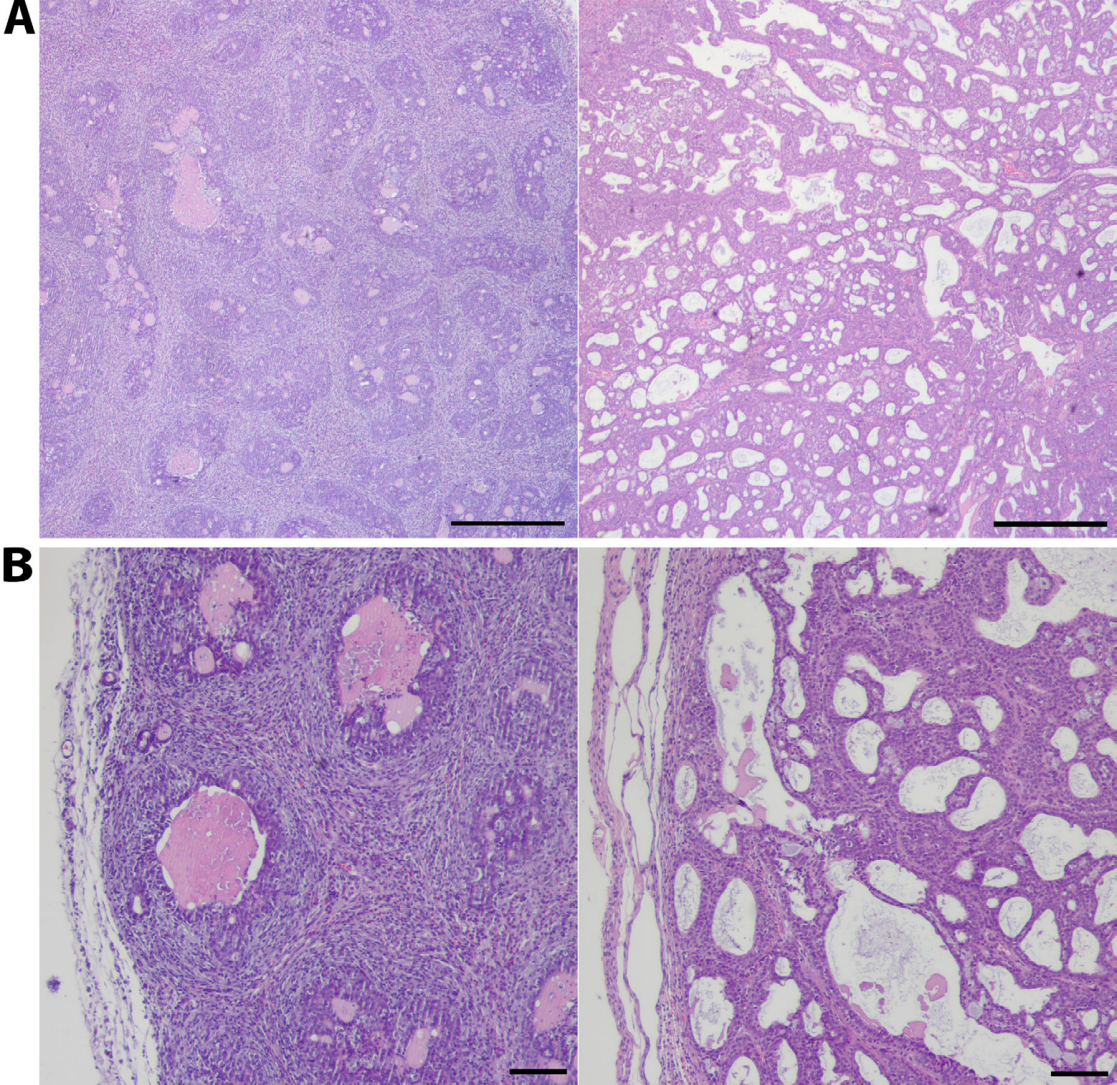
Examples of eosin-hematoxylin stained tumor-tissue of the central (A) and peripheral (B) part of the mammary tumor in control (left) and during hyperoxic treatment (right, 1 bar, pO_2 _= 1.0). The images under A are scaled to the same magnification (× 4) and the images under B to the same magnification (× 10). Scale bar indicate 500 μm (A) and 100 μm (B).

The gene expression data indicated that HBO treatment was associated with attenuation of glandular function in the mammary tumor. Subsequently the E & H stained tumor tissue sections, clearly demonstrates that normobaric and as well as hyperbaric hyperoxia treatment induced large areas of empty space ("vacuoles") both in the central parts (Fig 2A) and in the periphery (Fig 2B) of the tumor tissue compared to control (Table 3), thereby indicating a loss of glandular tissue. The morphology of 1 and 1.5 bar sections were identical.

### Tumor vessels

**Figure 3:**
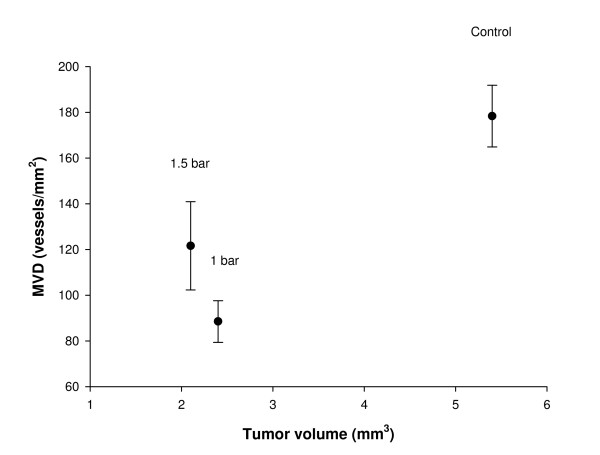
The relationship between tumor size and blood vessels per mm2 before and after hyperoxic treatment. Values represents mean ± SE.

In order to analyse the impact of hyperoxia on the induction of vessel formation, the vascular densities in "hot spot" areas (areas of high vascular density) in both the tumor centre and periphery were examined. The mean vascular density was markedly reduced in the tumors treated with hyperoxia compared to controls (Table 3). The ratio of central/peripheral vessel density is given in Table 3. The diameter of the remaining tumor vessels was, however, increased after hyperoxic treatment compared to control, as can be seen in Table 3. The results also show that the diameter of the peripheral vessels is larger than in the central parts in both control and at 1 bar hyperoxic treatment. The ratio of central/peripheral vessel diameter is given in Table 3. The relationship between tumor volume and mean vessel density is visualized in Fig 3.

### Tumor cells

The percentage of apoptotic cells in TUNEL-stained tissue section was significantly increased (18–21%) after hyperoxic treatment compared to controls as shown in Table 3.

## Discussion

The present study shows that exposing rats to normobaric (1 bar) or hyperbaric (1.5 bar) hyperoxia (100% O_2_), four times for 90 minutes, over a period of 10 days significantly retards tumor growth. In the present model this treatment showed more efficacy than the commonly used chemotherapeutic drug 5-FU. Hyperoxic treatment did affect the mammary tumors by significantly down-regulating glandular genes that may have implications in tumor growth (loss of glands), reducing vascular density and enhancing apoptosis.

### HBO is not a tumor promoter

HBO has been used in combination with both chemotherapy and radiotherapy to enhance the pO_2 _in the otherwise hypoxic tumor tissue and thereby potentiate the effect of these treatments [10]. HBO has also been used for wound healing, treatment of necrotic tissues and recovery of radiation-injured tissues because of its ability to promote angiogenesis and thereby enhance blood supply to the injured area [10,11]. However, it has for a long time been a misconception that HBO per se could have a tumor promoting effect. There are now several lines of evidence showing that this is not the case [13]. The present work support previous conclusions from our group demonstrating that HBO has a significant inhibitory effect on mammary tumor growth in rats [14]. Also two studies on mice injected with various tumor cell lines exposed to 70% O_2 _for 3 weeks showed a reduced number of lung colonies derived from mammary carcinoma MT-7 cells and of lung-tumor cell lines [17,18]. Some cell lines have, however been shown to be oxygen resistant [17], which indicates differences in the oxygen sensitivity in different tumor-types.

### Hyperoxia changes the morphology of the tumor by inducing loss of glands

In addition to the tumor growth retardation, the morphology of heamatoxylin-eosin stained tumor tissue showed significant changes after hyperoxic treatment. Most pronounced were the empty spaces in the tumor tissue, which suggests a loss of glands in the mammary tissue as indicated by a strong turn-off of glandular secretory proteins (parotid secretory protein, prolactin induced protein and common salivary protein-1) (Table 2). One of the glandular secretory proteins, prolactin, stimulates tumor growth and motility of human breast cancer cells and a recent review suggested that antagonizing prolactin should be evaluated as a tumor treatment [19]. It is therefore very surprising that hyperoxic treatment alone could down-regulate the prolactin induced protein and therby most likely also prolactin.

### Hyperoxia has an anti-angiogenic effect

Hypoxia induce the formation of new tumor blood vessels necessary for further tumor growth [20]. One might therefore expect that hyperoxia should prevent tumor angiogenesis. Normally, hyperoxic treatment is known to enhance angiogenesis in wound healing, in necrotic ulcers and after radiation injury [10,11]. Surprisingly, the present study demonstrates that hyperoxia reduce the mean vessel density in mammary tumors. This indicates that hyperoxia induce an anti-angiogenic effect in tumor tissue which is opposite to what is expected in "normal" tissues. Another, surprising finding was the increase in vascular diameter both in the periphery and central parts of the tumor, since HBO is generally know to induce vasoconstriction. This vasodilatory effect might indicate that the tumor is struggling to elevate its flow to compensate for the loss of blood vessels and thereby prevent starvation.

Many tumor models have demonstrated a close correlation between local oxygen deficiency and the production of HIF-1α and VEGF [21,22]. HIF-1α activity as well as VEGF secretion is tightly regulated by the oxygen-level, and in the well oxygenated state they are rapidly degraded [24]. Thus by increasing the pO_2 _in tumor tissue with normobaric and hyperbaric oxygen it is reasonable to assume that VEGF and HIF-1α would have been down-regulated. The anti-angiogenic effect found in the present study would correspond to this hypothesis.

### Hyperoxia induces apoptosis

We found the amount of normal cells (cells with intact nucleus) in the tumor to be decreased after hyperoxic treatment. It is therefore important to know that hyperoxic treatment in the present range (1 -1.5 bar, pO_2_= 1–1.5) and time exposed (90 min) have never been shown to induce cell death of other tissues of the body, including lungs which are known to be most susceptible to HBO (UHMS) [25]. A review by O'Reilly stated that cell proliferation (of normal tissue) in adult rats, mice and monkeys exposed to lethal levels of oxygen (>90%) was unaffected for the first 48, and alveolar epithelium relative unaffected until after 72 hours [26]. However, a recent report [27] on in vitro benign and malignant mammary epithelial cells showed that HBO (97.9% oxygen) at a high pressure (2.4 bar) inhibited epithelial cell proliferation. Furthermore, an organised, genetically programmed cell death was found in the tumor tissue after hyperoxic treatment as indicated by elevated amount of tunnel-positive apoptotic cells.

### Potential mechanism?

We might speculate that all or at least some of the changes we have mentioned after both normobaric and hyperbaric hyperoxia are due to free oxygen radicals (ROS). ROS activity is known to be elevated during hyperoxia [28,29]. When level of free oxygen radical production exceeds endogenous cellular antioxidant capacity it can create an "oxidative stress" which can lead to cell death [30]. Free oxygen radicals have previously been shown to induce apoptosis and also inhibit angiogenesis [31]. In the literature it seems as if ROS has "two faces". It appears as though the action of free radicals on normal and tumor cells are opposite. When free radicals attack normal cells, DNA damage can occur, whereas ROS in tumor cells has an unexpected, but highly beneficial action, namely inhibition of tumor cells [review:31]. Since the supoxide dismutase (SOD) activity in most tumors has been shown to be lower than in normal tissue, this might make the tumors even more susceptible to increased ROS activity during hyperoxic exposure. So, a moderate increase in ROS activity by HBO might be beneficial in tumors.

## Conclusion

The present study shows that both normobaric (1 bar, pO_2 _= 1.0) and hyperbaric hyperoxia (1.5 bar, pO_2_= 1.5) significantly retards mammary tumor growth in rats. Hyperoxic treatment did affect the mammary tumors by inducing loss of glands, reducing vascular density and enhancing apoptosis. The mechanisms behind these changes are still not known. Hyperbaric oxygen treatment should therefore be further evaluated as adjuvant tumor treatment.

## Competing interests

The author(s) declare that they have no competing interests.

## Authors' contributions

AR carried out the morphology, immunoassay (von willebrandt and Tunnel staining), statistical analysis and hyperbaric exposures.

CS and VMS carried out the gene expression profiling and PCR.

RB and RKR participated in the design and helped draft the manuscript.

LEBS conceived the study, supervised the hyperbaric exposures and wrote the manuscript.

All authors read and approved the final manuscript.

## Pre-publication history

The pre-publication history for this paper can be accessed here:



## Figures and Tables

**Table 1:** The experimental groups.

	**Gas**	**Total pressure (bar)**	**pO2 (bar)**	**Drug**	**No. of rats**
**Group 1**	Air	1.0	0.2	NaCl	10
**Group 2**	Air	1.0	0.2	5-FU	10
**Group 3**	Oxygen	1.0	1.0	NaCl	8
**Group 4**	Oxygen	1.5	1.5	NaCl	8

The hyperbaric oxygen treatement and 5-FU (0.2 mg/kg i.p.) were given on day 1, 4, 7 and 10.

**Table 2:** Hyperoxia-induced changes in expression of secretory proteins in DMBA-induced mammary tumors in rats

**Gene Name**	**Gene symbol**	**Change in expression level**^a^		**RefSeq**	**Probe ID**
				
		**Array**	**Q-PCR**		
*Parotid secretory protein*	Psp	3.0E-02	1.0E-04	NM_052808	A_43_P12746
*Prolactin induced protein*	Pip	5.1E-02	6.0E-04	NM_022708	A_43_P12308
*Cysteine-rich secretory protein 1*	Crisp1	4.6E-02	ND	NM_022859	A_42_P457387
*Common salivary protein 1*	Csp1	5.8E-02	7.1E-02	NM_133622	A_42_P562492
*Proline-rich proteoglycan 2*	Prpg2	2.2E-01	ND	NM_172065	A_42_P765648

^a) ^The fold change in expression level was determined as the ratio between HBO-treated- and control-tumors. Values correspond to fold decrease, i.e. down-regulation of expression. The data are means of ratios obtained from four HBO-treated tumors compared to a pool of control tumors. ND: Not determined.

**Table 3:** Area fraction of vacuoles, number of apoptotic cells, mean vascular density, vascular diameter and ratio central/peripheral vessel density and vessel diameter in controls and in tumors treated with hyperoxia. Values are given as means ± SD.

	**Control 1 bar, 21% O**_2 _ **pO**_2_** = 0.21**	**Test 1 bar, 100% O**_2 _ **pO**_2_** = 1.0**	**Test 1.5 bar, 100% O**_2 _ **pO**_2_** = 1.5**
Area fraction vacuoles "empty spaces"	0.01 ± 0.04	0.11 ± 0.05 ***	0.21 ± 0.02 ***
Apoptotic cells (% of total cells)	61% ± 6.4	85% ± 7.3 ***	88% ± 5.0 ***
Mean vascular density (number of vessels/mm^2^)			
Centrally	98 ± 18.6	50.1 ± 17.4***	69.8 ± 18.5*
Periphery	79.5 ± 19.2	38.5 ± 19.3***	51.8 ± 26.8*
Vascular diameter (μm)			
Centrally	19.1 ± 4.9	23.1 ± 9.1 **	23.2 ± 10.1**
Periphery	10.9 ± 3.9††	12.5 ± 4.7 *††	23.9 ± 10.0 ***
Ratio central/peripheral vessel density	1.2 ± 0.7	1.6 ± 1.3	1.5 ± 1.1*
Ratio central/peripheral vessel diameter	1.9 ± 0.8	2.0 ± 1.2	1.1 ± 0.6***

*P < 0.05, **P < 0.01, ***P < 0.001 vs control (1 bar, air)

††P < 0.01 vs centrally
